# U-shaped association between sleep duration and subjective cognitive complaints in Chinese elderly: a cross-sectional study

**DOI:** 10.1186/s12888-022-03738-0

**Published:** 2022-02-24

**Authors:** Li-Hua Lin, Wen-Qi Xu, Shi-Bin Wang, Qing Hu, Ping Zhang, Jia-Hao Huang, Yun-Fei Ke, Kai-Rong Ding, Cai-Lan Hou, Fu-Jun Jia

**Affiliations:** 1 School of Medicine, South China University of Technology, Guangzhou, 510006 Guangdong Province China; 2 Guangdong Mental Health Center, Guangdong Provincial People’s Hospital, Guangdong Academy of Medical Sciences, Guangzhou, 510080 Guangdong Province China; 3 School of Nursing, Southern Medical University, Guangzhou, 510515 Guangdong Province China; 4 Yuexiu District Center for Disease Control, Guangzhou, 510080 Guangdong Province China; 5 The Second School of Clinical Medicine, Southern Medical University, Guangzhou, 510515 Guangdong Province China

**Keywords:** U-shaped association, Sleep duration, Subjective cognitive decline, Subjective cognitive complaints, Generalized additive model

## Abstract

**Background:**

Subjective cognitive decline (SCD) may be the early screening signal to distinguish susceptible population with Alzheimer’s disease (AD) and mild cognitive impairment (MCI). Subjective cognitive complaints (SCCs) have been proved strongly associated with SCD. This study aimed to explore the association between sleep duration and SCCs in the Chinese elderly.

**Methods:**

We conducted a cross-sectional study involving 688 participants aged 60 years and older in Guangdong Province, China. SCCs were assessed by the Subjective Cognitive Decline questionnaire 9 (SCD-Q9), which contained 9 items with two dimensions, including the overall memory function and time comparison (OMTC) and daily activity ability (DAA). Restricted cubic splines and generalized additive model (GAM) were used to fit the association between sleep duration and SCD-Q9 score.

**Results:**

There were significant U-shaped associations between sleep duration and overall score of SCD-Q9 (*EDF* = 3.842, *P* < 0.001), as well as the OMTC dimension (*EDF* = 4.471, *P* < 0.001) in the age- and gender-adjusted GAM. The lowest points on the overall score of SCD-Q9 and OMTC score were observed in those sleeping 8 h per night. After further adjusting for other demographic characteristics, lifestyle behaviors, hypertension and diabetes, the U-shaped associations between sleep duration and the overall score of SCD-Q9 (*EDF* = 3.575, *P* = 0.004), sleep duration and the OMTC score (*EDF* = 4.478, *P* = 0.010) were still found. The daily activity ability (DAA) score was also non-linear associated with sleep duration both in the age- and gender-adjusted GAM (*EDF* = 2.314, *P* < 0.001) and further adjusted GAM (*EDF* = 2.080, *P* = 0.010).

**Conclusions:**

Both longer sleep duration (> 8 h) and shorter duration (< 8 h) were linked to worse SCCs. Future studies should explore the protective effect of managing sleep duration on SCD and its progression to dementia.

## Background

Data of the Seventh National Census of Populations of China showed that those 60 years or older were 18.7% of the national population and have increased by 5.44% compared to 2010 [1]. According to the standard of the United Nations (7%), China has already entered an aging society. Consequently, the increasing number of individuals suffering from cognitive impairment and dementia could lead to a serious burden for families, communities and health-care systems in China. China is the country with the largest number of dementias in the world and Alzheimer’s disease (AD) is the most popular type of dementia [2]. However, effective treatments for AD are still scarce. The treatments and interventions are required during the preclinical phase of the disease.

Subjective cognitive decline (SCD) is defined as the subjective cognitive decline, but without objective decline with cognitive measurement [3]. SCD may be the preclinical phase of AD and the early screening signal to distinguish susceptible population with AD and mild cognitive impairment (MCI) from general population [4]. According to previous studies, SCD was increasingly popular among Chinese elderly, with a prevalence of 14.4–18.8% in 2017 [5]. The incidence and prevalence of MCI and dementia in individuals with SCD are higher than those without SCD [6, 7]. Previous studies stated that the elderly suffering from SCD were twice as likely to have dementia as people without SCD [7]. And those with SCD developing MCI or dementia were 60% faster than normal people [8]. MCI and dementia may be prevented and delayed by managing SCD and its risk factors. Therefore, measuring the symptoms of SCD and its risk factors is conducive to early identification and intervention in the preclinical stage of AD and dementia, which is of great benefit for early prevention and public management of these diseases.

Before a clear definition of SCD was established, former studies usually focused on subjective cognitive complaints (SCCs), subjective cognitive impairment, subjective memory decline, subjective memory impairment, and memory complaints [9]. SCCs were considered as one of the diagnostic criteria for SCD and the SCD Initiative (SCD-I) stated that individuals with SCCs would be more susceptible to preclinical AD [3, 7]. SCCs refer to complaints about daily memory and related cognitive functions, regardless of whether there is objective cognitive impairment [7]. Although the associations between SCCs and objective cognitive decline are still controversial, SCCs have been considered as the risk factor for cognitive decline in most studies [10]. SCCs were found related to the reasons for objective cognitive decline, including greater brain atrophy and amyloid-beta (Aβ) deposition in the elderly [11, 12]. On the other hand, SCCs may reflect the susceptibility to psychological distress and negative effects that has been proved to the risk factor for AD in the elderly [10]. However, it is not flexible to screen SCD from the elderly due to the complex neuropsychological examinations in the general communities but simple and easy to assess SCCs using the self-rating scale [13]. Accordingly, it is of great importance to screen individuals with SCCs to identify those at high risk of SCD [14].

Sleep is manifested as weakened responsiveness to external environmental stimuli and disappearance in consciousness and active behavior for a certain period of time [15]. The National Sleep Foundation recommends that appropriate sleep duration of the elderly is 7 to 8 h for healthy individuals with normal sleep [16]. But many people continually deprived themselves of adequate sleep with increasing risk of accidents, physical and mental diseases, particularly in the elderly [17, 18]. Additionally, shorter sleep duration in normal cognitive function people was found related to greater cortical beta-amyloid burden, which was a precursor to cognitive declines [19]. Besides, longer sleep duration also showed strong association with negative outcomes including objective cognitive decline, physical and mental health [20–22]. Therefore, short and long sleep duration are increasingly considered as an effective factor of health in modern society [23, 24]. It is significant to discern the dangers behind the short or long sleep duration.

In the past years, most studies focused on memory complaints while ignoring other areas of cognition and demonstrated that there was a non-linear correlation between sleep duration and objective cognitive decline [25, 26]. It was found that individuals with short sleep duration or long sleep duration tended to report worse memory complaints [25, 27]. Apart from memory, few studies have focused on other cognitive domains such as executive function. Additionally, there are no studies to test the association between sleep duration and SCCs, which are strongly correlated with SCD. The association between sleep duration and SCCs merit further study.

Accordingly, this study aimed to explore the association between sleep duration and the SCCs, which involved the overall memory function and time comparison, and the daily activity ability. Moreover, this study could provide important information about the profile of SCCs in the Chinese elderly population and may be of special relevance for the general practice to screen for individuals at risk of SCD.

## Methods

### Study population and procedures

Our study was based on the Guangzhou Sleep and Psychosomatic Health Survey, which was a face-to-face health interview and physical examination survey conducted from November 2020 to March 2021. A total of 688 elderly living in Guangzhou were recruited from the 7 communities and 2 nursing homes. Inclusion criteria include: (1) ≥ 60 years old; (2) Being able to take part in a face-to-face interview. Exclusion criteria include: (1) < 60 years old; (2) Self-reported with neurological disease, involving Alzheimer’s disease (AD), Parkinson’s disease, brain atrophy, stroke, epilepsy, sleep disorders, anxiety disorder and depression disorder. (3) Being unable to complete the interview.

The study was conducted in accordance with the Declaration of Helsinki and approved by the Research Ethics Committee of the Guangdong Provincial People’s Hospital, Guangdong Academy of Medical Sciences (Reference number: GDREC2018543H (R1)). All participants have provided written informed consent to accept the interview.

### Data collection and measurement

Standardized questionnaires were applied to collect information about demographic characteristics, lifestyle variables (smoking, alcohol consumption, tea drinking, physical activity, and diet), chronic diseases status (hypertension, diabetes, hyperlipidemia, coronary heart disease, neurological disease) as well as mental health status (anxiety and depression). All the obtained information was imported into the database using the ‘Questionnaire Star’ electronic questionnaire system.

### Sleep duration

Sleep duration was recorded by asking the following question “In the past month, how long did you actually sleep at night?”. Based on earlier epidemiological data in Guangdong, the elderly was divided into three groups according to reported sleep duration (< 6 h, 6 to 8 h, and ≥ 8 h), and those who sleep 6 to 8 h were set to be the reference group [28].

### Subjective cognitive complaints

SCCs were assessed by the SCD questionnaire 9 (SCD-Q9), which was regarded as a credible scale to evaluate the complaints of cognitive decline. Cronbach’s alpha of reliability was 0.847 and the coefficient of validity was 0.871 for the translated version of SCD-Q9 used in screening subjective cognitive decline in Chinese elderly [5]. SCD-Q9 contains 9 items with two dimensions, including the overall memory function and time comparison (OMTC, 4 items) and daily activity ability (DAA, 5 items). SCD-Q9 includes questions about global memory functioning, temporal comparisons, and ability to finish daily or routine activities [29]. The total score of SCD-Q9 varies from 0 to 9, with a greater score suggesting worse complaints of cognition [30]. In our study, Cronbach’s alpha was 0.78 for SCD-Q9.

### Socio-demographic and lifestyle factors

The age of participants was defined as two groups: ≤ 70 years old and > 70 years old [31]. Residence was classified as nursing home and community. Education level was categorized as four groups: primary school or lower, junior high school, senior high school, and college or higher. Cohabitation status referred to whether or not to live with others. Monthly income was divided into three groups: < 3500 yuan, 3500 to 5999 yuan, and ≥ 6000 yuan. Current smoking meant smoking at least 1 cigarette per day currently and continuing smoking for 6 months or more. Alcohol consumption meant drinking at least once per week. Tea-drinking habit meant drinking tea at least four times a week, including green tea, oolong tea, and black tea. Physical exercise frequency was divided into three groups: hardly or never exercise, occasionally exercise (1–2 times a week or less), frequently exercise (3 times a week or more). Body mass index (BMI) was defined as weight in kilograms divided by height in meters squared. According to the reference standard for the Chinese population, BMI was divided into three groups: < 18.5 kg/m^2^, 18.5 to 24.0 kg/m^2^, and ≥ 24 kg/m^2^ [32]. The napping habit was defined as having napped at least once in the past week and obtained through the following questions: “Do you take a nap during the daytime? If yes, how long (minutes) is the nap duration?”

### Mental health status

Anxiety symptoms were assessed by the 7-item Generalized Anxiety Disorder module (GAD-7). The total score of the GAD-7 varies from 0 to 21 and score ≥ 5 means to suffer anxiety symptoms [33]. The Cronbach’s alpha for the internal consistency reliability of the GAD-7 was 0.83 for the entire scale in this study. Depressive symptoms were measured using the 9-item Patient Health Questionnaire (PHQ-9). PHQ-9 score ≥ 5 represented suffering depression symptoms [34]. The Cronbach’s alpha for the internal consistency reliability of the PHQ-9 was 0.71 for the entire scale in this study.

More details of the survey setting can be found in other survey report [35].

### Statistical analyses

Generalized additive models (GAM) is an extension of the generalized linear model which allows the evaluation for the non-linear association of the outcome and the predictors without prior knowledge of the relationship between variables [36]. Model assumptions were assessed by investigating the normality of residuals, homoscedasticity, and residual symmetry. The non-linear association of sleep duration and SCD-Q9 score was measured through generalized additive models (GAM). Restricted cubic splines were fitted to the association between target variables. The *effective degree of freedom* (*EDF*) value in GAM indicated the amount of non-linearity of the smooth. *EDF* value of 1 suggests linear association between target variables and value of EDF > 1 indicates a more complex relationship between SCCs and sleep duration. Both SCD-Q9 score and sleep duration have been found abnormal distribution based on the Kolmogorov-Smirnov test (*P* < 0.001). Therefore, differences among short (< 6 h), normative (6–8 h) and long sleepers (≥ 8 h) were performed by the Mann-Whitney U test and Kruskal-Wallis test. The different score of SCD-Q9 and its two dimensions among different groups were also tested by the Mann-Whitney U test and Kruskal-Wallis test. Statistical analyses were carried out using SPSS (IBM SPSS, version 26.0) and R (version 3.6.0). Two-tailed *P* value of < 0.05 shows statistically significant.

## Results

A total of 688 participants were involved in the analysis with the mean age of 73.79 years old (SD =8.28, range: 60–101). Among the 688 participants, 243 participants (35.3%) slept for less than 6 h, 356 participants (51.7%) slept for 6 to 8 h, and 89 participants (12.9%) slept for 8 h or more. Table 1 represented the comparison between short (< 6 h), normative (6–8 h) and long sleepers (≥ 8 h) by socio-demographic characteristics, lifestyle factors, common chronic diseases status and mental health. Sleep duration was significantly different in terms of age, gender, residence, cohabitation status, current smoking, tea-drinking habit, hypertension, depression symptoms and anxiety symptoms (*P* < 0.05). Table 2 showed the different score of SCD-Q9 and its two dimensions among different groups. Age, gender, residence, education level, cohabitation status, monthly income, current smoking, alcohol consumption, tea-drinking habit, BMI, napping habit, depression symptoms and anxiety symptoms were significantly associated with the overall score of SCD-Q9 (*P < 0.05*).

**Table 1:** Characteristics of participants by sleep duration per day^a^

Variables	Groups	< 6 h (*n* = 243)	6–8 h (*n* = 356)	≥ 8 h (*n* = 89)	*P*
Age (years)	≤ 70(%)	86(35.4)	150(42.1)	49(55.1)	0.002
	> 70(%)	157(64.6)	206(57.9)	40(44.9)	
Gender	Male(%)	74(30.5)	140(39.3)	45(50.6)	0.001
	Female(%)	169(69.5)	216(60.7)	44(49.4)	
Residence	Community(%)	143(58.8)	249(69.9)	71(79.8)	< 0.001
	Nursing home(%)	100(41.2)	107(30.1)	18(20.2)	
Education level	Primary school or lower(%)	67(27.6)	76(21.3)	14(15.7)	0.088
	Junior high school(%)	63(25.9)	101(28.4)	22(24.7)	
	Senior high school(%)	77(31.7)	114(32.0)	38(42.7)	
	College or higher(%)	36(14.8)	65(18.3)	15(16.9)	
Cohabitation status	Married/Cohabitant(%)	133(54.7)	242(68.0)	72(80.9)	0.001
Monthly income (yuan)	< 3500(%)	42(17.3)	75(21.1)	26(29.2)	0.057
	3500 to 5999(%)	151(62.1)	210(59.0)	53(59.6)	
	> 6000(%)	50(20.6)	71(19.9)	10(11.2)	
Current smoking	Yes(%)	15(6.2)	38(10.7)	12(13.5)	0.021
Alcohol consumption	Yes(%)	13(5.3)	25(7.0)	9(10.1)	0.146
Tea-drinking habits	Yes(%)	73(30.0)	154(43.3)	44(49.4)	< 0.001
Exercise frequency	Hardly(%)	42(17.3)	67(18.8)	13(14.6)	0.199
	Occasionally(%)	19(7.8)	42(11.8)	12(13.5)	
	Frequently(%)	182(74.9)	247(69.4)	64(71.9)	
BMI (kg/m^2^)	< 18.5(%)	22(9.1)	26(7.3)	7(7.9)	0.822
	18.5 to 24(%)	123(50.6)	188(52.8)	45(50.6)	
	≥ 24(%)	98(40.3)	142(39.9)	37(41.6)	
Napping habit	Yes(%)	189(77.8)	277(77.8)	71(79.8)	0.781
Hypertension	Yes(%)	155(63.8)	190(53.4)	49(55.1)	0.026
Diabetes	Yes(%)	51(21.0)	78(21.9)	24(27.0)	0.346
Depression symptoms	Yes(%)	64(26.3)	29(8.1)	2(2.2)	< 0.001
Anxiety symptoms	Yes(%)	18(7.4)	15(4.2)	2(2.2)	0.030

*BMI* Body mass index

^a^Data were statistically analyzed using the Mann-Whitney U test and Kruskal-Wallis test

**Table 2:** Characteristics of participants by SCD-Q9 score^a^

Variables	Groups	Overall score of SCD-Q9		Overall memory function and time comparison score		Daily activity ability score	
		*Median(Q1,Q3)*	*P*	*Median(Q1,Q3)*	*P*	*Median(Q1,Q3)*	*P*
Age (years)	≤ 70	3.00(1.00,5.00)	< 0.001	2.00(1.00,3.00)	< 0.001	1.0(0.00,2.50)	< 0.001
	> 70	4.50(2.50,6.00)		3.00(1.00,4.00)		1.5(0.50,2.50)	
Gender	Male	3.00(1.00,5.00)	< 0.001	2.00(0.00,3.00)	< 0.001	1.00(0.00,1.50)	< 0.001
	Female	4.50(2.50,6.00)		3.00(1.00,4.00)		2.50(0.50,2.50)	
Residence	Community	3.00(1.00,5.00)	< 0.001	3.00(1.00,4.00)	< 0.001	1.00(0.00,1.50)	< 0.001
	Nursing home	5.50(3.50,6.50)		3.00(2.00,4.00)		2.00(1.00,2.50)	
Education level	Primary school or lower	5.50(3.50,6.50)	< 0.001	3.00(2.00,4.00)	0.001	2.00(1.00,3.00)	< 0.001
	Junior high school	3.50(1.00,6.00)		3.00(1.00,4.00)		1.00(0.00,2.50)	
	Senior high school	3.50(1.00,5.50)		3.00(1.00,4.00)		1.00(0.00,2.00)	
	College or higher	4.00(1.50,5.50)		3.00(1.00,4.00)		1.00(0.125,2.00)	
Cohabitation	Married/Cohabitant	3.50(1.00,5.50)	< 0.001	3.00(1.00,4.00)	< 0.001	1.00(0.00,2.00)	< 0.001
	Widowed/separated/single	5.00(2.50,6.50)		3.00(1.00,4.00)		1.50(1.00,2.50)	
Monthly income (yuan)	< 3500	3.50(1.00,6.00)	0.009	2.00(0.00,4.00)	0.001	1.50(0.50,2.50)	0.025
	3500 to 5999	4.00(1.50,5.50)		3.00(1.00,4.00)		1.00(0.00,2.00)	
	≥ 6000	5.00(3.00,6.00)		2.00(1.00,4.00)		1.50(0.50,2.50)	
Current smoking	Yes	1.50(0.00,3.75)	< 0.001	1.00(0.00,4.00)	< 0.001	0.50(0.00,1.50)	< 0.001
	No	4.00(2.00,6.00)		3.00(1.00,4.00)		1.50(0.50,2.50)	
Alcohol consumption	Yes	2.50(0.50,4.50)	0.002	1.00(0.00,3.00)	0.009	0.50(0.00,1.50)	0.003
	No	4.00(1.50,6.00)		3.00(1.00,4.00)		1.50(0.50,2.50)	
Tea-drinking habit	Yes	3.00(1.00,5.50)	< 0.001	2.00(0.00,4.00)	< 0.001	1.00(0.00,2.00)	< 0.001
	No	4.50(2.00,6.00)		3.00(1.00,4.00)		1.50(0.50,2.50)	
Exercise frequency	Hardly	4.00(1.00,6.00)	0.960	3.00(1.00,4.00)	0.924	1.00(0.375,2.50)	0.910
	Occasionally	4.00(1.00,6.50)		3.00(1.00,4.00)		1.00(0.00,2.50)	
	Frequently	4.00(1.50,5.50)		3.00(1.00,4.00)		1.50(0.50,2.00)	
BMI (kg/m^2^)	< 18.5	5.50(3.00,6.50)	0.043	3.00(1.00,4.00)	0.154	1.50(1.00,2.50)	0.026
	18.5 to 24	4.00(1.50,6.00)		3.00(1.00,4.00)		1.00(0.50,2.375)	
	≥ 24	4.00(1.50,5.50)		3.00(1.00,4.00)		1.00(0.25,2.00)	
Napping habit	Yes	4.50(1.50,6.00)	0.002	3.00(1.00,4.00)	< 0.001	1.50(0.25,2.50)	0.030
	No	3.00(1.00,5.00)		2.00(1.00,3.00)		1.00(0.00,2.00)	
Hypertension	Yes	4.00(1.87,6.00)	0.250	3.00(1.00,4.00)	0.165	1.00(0.00,2.125)	0.576
	No	3.50(1.00,6.00)		3.00(1.00,4.00)		1.5(0.50_,2.125)	
Diabetes	Yes	4.50(2.00,6.00)	0.166	3.00(1.00,4.00)	0.241	1.50(0.50,2.50)	0.200
	No	4.00(1.50,5.50)		3.00(1.00,4.00)		1.00(0.50,2.00)	
Depression symptoms	Yes	5.50(4.00, 7.00)	< 0.001	4.00(3.00,4.00)	< 0.001	2.00(1.00,3.00)	< 0.001
	No	3.50(1.00,5.50)		3.00(1.00,4.00)		1.00(0.00,2.00)	
Anxiety symptoms	Yes	6.00(4.50,7.00)	< 0.001	4.00(2.00,4.00)	0.002	2.00(1.50,3.50)	< 0.001
	No	4.00(1.50,5.50)		3.00(1.00,4.00)		1.00(0.50,2.00)	

*Q1* Lower Quartile, *Q3* Upper Quartile, *SCD-Q9* 9-items subjective cognitive decline questionnaire, *BMI* Body mass index

^a^Data were statistically analyzed using the Mann-Whitney U test and Kruskal-Wallis test

**Fig. 1:**
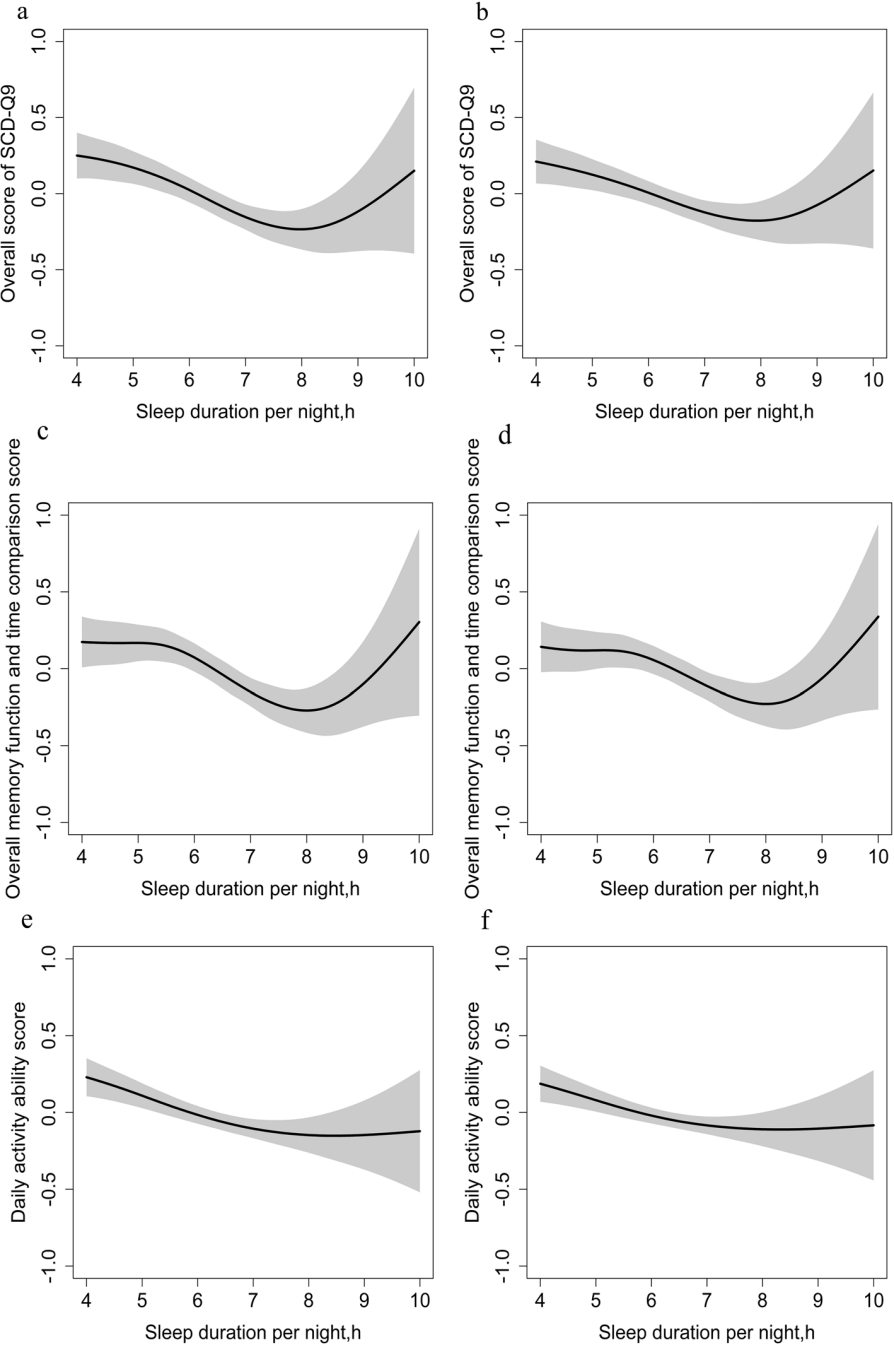
Associations between sleep duration per night and overall score of SCD-Q9 (**a**) (**b**), the overall memory function and time comparison score (**c**) (**d**), and daily activity ability score (**e**) (**f**) respectively. The **a**, **c**, **e** adjust for age and gender; The **b**, **d**, **f** adjust for age and gender, residence, education, married/ cohabitant status, monthly income, current smoking status, alcohol consumption, tea-drinking habit, napping habit, exercise frequency, BMI, hypertension and diabetes

Table 3 revealed the results of multivariable GAM for the association of SCD-Q9 score and selected independent variables. After adjusting for age and gender, a significant U-shaped association between sleep duration and overall score of SCD-Q9 (*EDF* = 3.842, *P* < 0.001), as well as OMTC score (*EDF* = 4.471, *P* < 0.001) was found. The U-shaped association between sleep duration and overall score of SCD-Q9 (*EDF* = 3.575, *P* = 0.004), as well as OMTC score (*EDF* = 4.478, *P* = 0.010) still existed after further adjusting for residence, education, cohabitation status, monthly income, current smoking, alcohol consumption, tea-drinking habit, napping habit, exercise frequency, BMI, hypertension and diabetes. Those participants who slept for 8 h had the lowest score of overall score of SCD-Q9 and OMTC score (Fig. 1). The association between sleep duration and DAA score did not show an obvious U shape (Fig. 1). But the obvious non-linear association between sleep duration and DAA score existed in the age- and sex-adjusted model (*EDF* = 2.314, *P* = 0.001). After further adjusting for other socio-demographic characteristics, lifestyle factors and common chronic diseases status, a significant non-linear correlation was still observed between sleep duration and DAA score (*EDF* = 2.080, *P* = 0.011).

**Table 3:** Association of SCD-Q9 score and independent variables measured by the multivariable generalized additive model

Variables	Overall score of SCD-Q9				Overall memory function and time comparison score				Daily activity ability score			
	Model1^a^		Model2^b^		Model1^a^		Model2^b^		Model1^a^		Model2^b^	
	*B*	*p*	*B*	*p*	*B*	*p*	*B*	*p*	*B*	*p*	*B*	*p*
Age(> 70 years)	0.435	< 0.001	0.176	0.036	0.366	< 0.001	0.142	0.101	0.405	< 0.001	0.171	0.042
Gender (Female)	0.478	< 0.001	0.350	< 0.001	0.452	< 0.001	0.339	< 0.001	0.387	< 0.001	0.271	0.001
Residence (Nursing home)			0.297	0.002			0.113	0.259			0.450	< 0.001
Education (vs. Primary school or lower)												
Junior high school			−0.261	0.011			−0.233	0.029			− 0.228	0.027
Senior high school			−0.324	0.002			−0.237	0.027			− 0.342	0.001
College or higher			−0.264	0.037			−0.196	0.135			−0.280	0.028
Married/ Cohabitant (yes)			0.030	0.719			−0.031	0.716			0.095	0.248
Monthly income (vs. Less than 3500)												
3500–5999			0.057	0.541			0.200	0.036			−0.140	0.130
≥ 6000			0.118	0.383			0.345	0.014			−0.205	0.130
Current smokers (yes)			0.313	0.022			0.345	0.015			0.186	0.172
Alcohol consumption (yes)			−0.020	0.890			−0.084	0.578			0.063	0.667
Tea-drinking habits (yes)			0.113	0.140			0.165	0.037			0.019	0.804
Napping habits (yes)			0.185	0.035			0.228	0.012			0.083	0.345
Exercise frequency (vs. Hardly)												
Frequently			−0.023	0.867			−0.082	0.559			0.056	0.677
Occasionally			−0.135	0.151			−0.077	0.428			−0.175	0.065
BMI (kg/m^2^) (vs. < 18.5)												
18.5 to 24			−0.243	0.068			−0.248	0.071			−0.168	0.208
≥ 24			−0.240	0.080			−0.230	0.104			−0.186	0.176
Hypertension (yes)			0.0004	0.995			0.041	0.578			−0.049	0.494
Diabetes (yes)			0.087	0.303			0.080	0.361			0.073	0.391
Sleep duration (h/night)	Smooth Curve, *EDF* = 3.842	< 0.001	Smooth Curve, *EDF* = 3.575	0.004	Smooth Curve, *EDF* = 4.471	< 0.001	Smooth Curve, *EDF* = 4.478	0.010	Smooth Curve, *EDF* = 2.314	0.001	Smooth Curve, *EDF* = 2.080	0.010

*SCD-Q9* 9-items subjective cognitive decline questionnaire, *BMI* Body mass index

^a^Model 1: Adjusted for age and gender

^b^Model2: Adjusted for age and gender, residence, education, married/ cohabitant status, monthly income, current smoking status, alcohol consumption, tea-drinking, napping habits, exercise frequency, BMI, hypertension, diabetes

The associations between sleep duration and overall score of SCD-Q9 (*EDF* = 3.689, *P* = 0.158), sleep duration and OMTC score (*EDF* = 4.464, *P* = 0.136), as well as sleep duration and DAA score (*EDF* = 1.907, *P* = 0.147) was no longer significant after further adjusting for depression and anxiety symptoms.

## Discussion

Our study investigated the association between sleep duration and SCCs among Chinese elderly. A statistically significant U-shaped association between sleep duration and SCCs was found in the models after adjusting for socio-demographic characteristics, lifestyle factors and common chronic diseases status. Both longer sleep duration (> 8 h) and shorter duration (< 8 h) were related to the worse SCCs. It was the first paper to demonstrate the U-shaped association between sleep duration and SCCs, as well as its two dimensions. This study filled the gap in the correlation between sleep duration and SCCs. These results could provide more information about the profile of SCCs in the Chinese elderly.

The associations between sleep duration and SCCs were not statistically significant after adjusting for depression symptoms and anxiety symptoms. This result suggested anxiety symptoms and depression symptoms played important roles in the association between sleep and SCCs. For example, anxiety symptoms and depression symptoms may exist mediating effect or moderating effect in the association between sleep duration and SCCs, which was consistent with earlier results [35]. Further studies are needed to prove it.

Earlier study found a non-linear association between cognitive impairment and sleep duration [25, 37], which was similar to our findings. A longitudinal analysis based on women showed a V-shaped association between higher MCI/dementia risk and sleep duration, indicating both shorter (< 6 h) and longer (> 8 h) sleep duration associated with higher risk of cognitive decline [26]. Additionally, those with shorter and longer sleep duration have worse memory complaints in general [25]. Another longitudinal study found that a U-shaped association between sleep duration and global cognitive decline existed among people aged 45 years or older, indicating that people sleeping both less than 4 h and more than 10 h appeared worse cognition function and faster impairment in both global cognitive function and memory [21]. The difference in sleep duration might be caused by differences in research population and cognitive dimensions.

SCD is related to neuropathological changes linking to AD pathogenesis involving the elevated amyloid-β deposition, increased white matter lesions, smaller left hippocampal volumes, and temporal lobe atrophy [38–40]. The biological mechanisms linking sleep duration to cognitive impairment are not identified. Sleep duration has been proved to relate to multiple factors including physical health status, mental health status, living conditions and so on [28, 41]. Getting clear the reasons inducing shorter or longer sleep duration is critical for researchers to comprehend the U-shaped association between sleep duration and cognitive decline.

Short sleep duration and sleeping fragmentation are very common among the elderly [42, 43]. Short sleep duration can be explained as prolonged awaking or lack of sleep [44]. Adequate sleep duration is vital to the formation and consolidation of memory [45]. Short sleep duration would weaken cognitive performance by promoting the deposition of amyloid and tau, and inflammation [46]. According to previous study, shortening of sleep duration would lead to the increase in tumor necrosis factor α(TNFα) level and then promoted inflammation [47]. In addition, metabolites in the brain were eliminated during sleeping. Short sleep duration would cause not only the reduction for hippocampal synthesis of proteins and impair hippocampal neurogenesis, but also increase the deposition of adenosine and Aβ which damaged synapse plasticity [44].

On the other hand, the reasons why longer sleep duration associated with worse cognitive impairment and SCCs are still unclear. Former studies found that individuals with AD stayed awake longer in bed and occurred more fragmented sleep than those without AD [46]. Longer sleep duration may point out physical diseases, sleep disorders or related diseases, drug effects, and frailty [18, 48, 49]. Some studies claimed that those with longer sleep duration reflected a proinflammatory state associated with the pathophysiology of AD [50, 51]. The increase in sleep duration pointed out the rise of inflammatory markers involving C-reactive protein (CRP) andinterleukin-6 (IL-6). For every hour extension of sleep duration, CRP levels would elevate by 8 and 7% IL-6 levels would increase by 7% [47]. Inflammatory might also be the biological mechanism linking longer sleep duration and SCD. In addition to inflammatory, those with longer sleep duration tended to change daily rest–activity rhythm related to AD and PD [52, 53]. More mechanism studies are needed to explore whether long sleep duration is a subtle marker of cognitive decline in the healthy elderly.

Poor global memory functioning questions were linked to cognitive performances [54]. In our study, the association between sleep duration and OMTC score also appeared U-shaped. But the U-shaped relationship was weakened and closer to linear in the association between sleep duration and DAA score. Those results could be explained when there were complaints about memory, people with SCD may still have the normal ability of daily living.

This study was conducted on a very large sample and many variables to explore the association between sleep duration. Additionally, this study was feasible to assess SCCs by SCD-Q9 among older adults. There were also some limitations to the present study. First, same with other cross-sectional studies, effects from unmeasured variables such as medication use could not be excluded and causality between SCCs and sleep duration could not be examined. Secondly, rather than obtaining from accurate measurement by objective methods, information about sleep duration was from participant self-reported. However, it was unrealistic to utilize objective methods (like polysomnography) to assess the sleep duration in such a large sample study, which was a common limitation of sleep-related epidemiological studies. Thirdly, it was only a symptomatic study toward SCCs due to the lack of other neuropsychological examinations. Further screening tools such as Mini-mental State Examination and Montreal Cognitive Assessment were needed to diagnose SCD. Fourthly, the participants were only from one city in south China, which would weaken the representation of study results in the Chinese population. Despite the above limitations, this study did fill the gap in the correlation between sleep duration and SCCs. Moreover, this study could provide more information about the profile of SCCs in the Chinese elderly.

## Conclusions

There was a U-shaped association between SCCs and sleep duration. Both longer sleep duration (> 8 h) and shorter duration (< 8 h) were linked to worse SCCs. Future studies should explore the protective effect of managing sleep duration on SCD and its progression to dementia.

## Data Availability

The datasets used during the current study are available from the corresponding author on reasonable request.

## Abbreviations

AD: Alzheimer’s disease
MCI: Mild cognitive decline
SCD: Subjective cognitive decline
SCCs: Subjective cognitive complaints
SCD-Q9: 9-items subjective cognitive decline questionnaire
PHQ-9: Patient Health Questionnaire-9
GAD-7: Generalized Anxiety Disorder-7
GAM: Generalized additive model
BMI: Body mass index
EDF: Effective degree of freedom
OTMC: Overall memory function and time comparison
DAA: Daily activity ability
CRP: C-reactive protein
IL-6: Interleukin-6

## References

[1] National Bureau of Statistics (2021). Communiqué of the seventh National Census of China.

[2] Jia L, Quan M, Fu Y, Zhao T, Li Y, Wei C, Tang Y, Qin Q, Wang F, Qiao Y (2020). Dementia in China: epidemiology, clinical management, and research advances. Lancet Neurol.

[3] Jessen F, Amariglio RE, van Boxtel M, Breteler M, Ceccaldi M, Chételat G, Dubois B, Dufouil C, Ellis KA, van der Flier WM (2014). A conceptual framework for research on subjective cognitive decline in preclinical Alzheimer's disease. Alzheimers Dement.

[4] Fernández-Blázquez MA, Ávila-Villanueva M, Maestú F, Medina M (2016). Specific features of subjective cognitive decline predict faster conversion to mild cognitive impairment. J Alzheimers Dis.

[5] Hao L, Wang X, Zhang L, Xing Y, Guo Q, Hu X, Mu B, Chen Y, Chen G, Cao J (2017). Prevalence, risk factors, and complaints screening tool exploration of subjective cognitive decline in a large cohort of the Chinese population. J Alzheimers Dis.

[6] Slot RER, Sikkes SAM, Berkhof J, Brodaty H, Buckley R, Cavedo E, Dardiotis E, Guillo-Benarous F, Hampel H, Kochan NA (2019). Subjective cognitive decline and rates of incident Alzheimer's disease and non-Alzheimer's disease dementia. Alzheimers Dement.

[7] Mitchell AJ, Beaumont H, Ferguson D, Yadegarfar M, Stubbs B (2014). Risk of dementia and mild cognitive impairment in older people with subjective memory complaints: meta-analysis. Acta Psychiatr Scand.

[8] Reisberg B, Shulman MB, Torossian C, Leng L, Zhu W (2010). Outcome over seven years of healthy adults with and without subjective cognitive impairment. Alzheimers Dement.

[9] Abdulrab K, Heun R (2008). Subjective memory impairment. A review of its definitions indicates the need for a comprehensive set of standardised and validated criteria. Eur Psychiatry.

[10] Snitz BE, Weissfeld LA, Cohen AD, Lopez OL, Nebes RD, Aizenstein HJ, McDade E, Price JC, Mathis CA, Klunk WE (2015). Subjective cognitive complaints, personality and brain amyloid-beta in cognitively Normal older adults. Am J Geriatr Psychiatry.

[11] Cedres N, Machado A, Molina Y, Diaz-Galvan P, Hernández-Cabrera JA, Barroso J, Westman E, Ferreira D (2019). Subjective cognitive decline below and above the age of 60: a multivariate study on neuroimaging, cognitive, clinical, and demographic measures. J Alzheimers Dis.

[12] Amariglio RE, Becker JA, Carmasin J, Wadsworth LP, Lorius N, Sullivan C, Maye JE, Gidicsin C, Pepin LC, Sperling RA (2012). Subjective cognitive complaints and amyloid burden in cognitively normal older individuals. Neuropsychologia.

[13] Yu S, Xiaoni W, Guanqun C, Can S, Xuanyu L, Qin Y, Taoran L, Wenying D, Xiaoqi W, Li L (2020). Interpretation of subjective cognitive decline characteristics published in lancet neurology. Chin J Neurol.

[14] Dumas JA (2021). Subjective cognitive complaints in aging: a commentary on Topiwala et al. 2020. Am J Geriatr Psychiatry.

[15] Rasch B, Born J (2013). About sleep's role in memory. Physiol Rev.

[16] Hirshkowitz M, Whiton K, Albert SM, Alessi C, Bruni O, DonCarlos L, Hazen N, Herman J, Katz ES, Kheirandish-Gozal L (2015). National Sleep Foundation's sleep time duration recommendations: methodology and results summary. Sleep Health.

[17] Zhai L, Zhang H, Zhang D (2015). Sleep duration and depression among adults: a meta-analysis of prospective studies. Depress Anxiety.

[18] St-Onge MP, Grandner MA, Brown D, Conroy MB, Jean-Louis G, Coons M, Bhatt DL (2016). Sleep duration and quality: impact on lifestyle behaviors and Cardiometabolic health: a scientific statement from the American Heart Association. Circulation.

[19] Bateman RJ, Xiong C, Benzinger TL, Fagan AM, Goate A, Fox NC, Marcus DS, Cairns NJ, Xie X, Blazey TM (2012). Clinical and biomarker changes in dominantly inherited Alzheimer's disease. N Engl J Med.

[20] Wang C, Bangdiwala SI, Rangarajan S, Lear SA, AlHabib KF, Mohan V, Teo K, Poirier P, Tse LA, Liu Z (2019). Association of estimated sleep duration and naps with mortality and cardiovascular events: a study of 116 632 people from 21 countries. Eur Heart J.

[21] Ma Y, Liang L, Zheng F, Shi L, Zhong B, Xie W (2020). Association between sleep duration and cognitive decline. JAMA Netw Open.

[22] Swinkels CM, Ulmer CS, Beckham JC, Buse N, Calhoun PS (2013). The Association of Sleep Duration, mental health, and health risk behaviors among U.S. Afghanistan/Iraq Era veterans. Sleep.

[23] Liew SC, Aung T (2021). Sleep deprivation and its association with diseases- a review. Sleep Med.

[24] Pizinger TM, Aggarwal B, St-Onge MP (2018). Sleep extension in short sleepers: An evaluation of feasibility and effectiveness for weight management and Cardiometabolic disease prevention. Front Endocrinol (Lausanne).

[25] Kronholm E, Sallinen M, Suutama T, Sulkava R, Era P, Partonen T (2009). Self-reported sleep duration and cognitive functioning in the general population. J Sleep Res.

[26] Chen JC, Espeland MA, Brunner RL, Lovato LC, Wallace RB, Leng X, Phillips LS, Robinson JG, Kotchen JM, Johnson KC (2016). Sleep duration, cognitive decline, and dementia risk in older women. Alzheimers Dement.

[27] Gamaldo AA, Wright RS, Aiken-Morgan AT, Allaire JC, Thorpe RJ, Whitfield KE (2019). The association between subjective memory complaints and sleep within older African American adults. J Gerontol B Psychol Sci Soc Sci.

[28] Chen X, Wang SB, Li XL, Huang ZH, Tan WY, Lin HC, Hou CL, Jia FJ (2020). Relationship between sleep duration and sociodemographic characteristics, mental health and chronic diseases in individuals aged from 18 to 85 years old in Guangdong province in China: a population-based cross-sectional study. BMC Psychiatry.

[29] Hao L, Hu X, Han Y, Jia J. The Sinicization and reliability and validity analysis of the English version of subjective cognitive decline questionnaire. Chinese Gen Pract. 2019;26(22):3238–3245

[30] Hao L, Sun Y, Li Y, Wang J, Wang Z, Zhang Z, Wei Z, Gao G, Jia J, Xing Y (2020). Demographic characteristics and neuropsychological assessments of subjective cognitive decline (SCD) (plus). Ann Clin Transl Neurol.

[31] Liu X, Chen J, Geng R, Wei R, Xu P, Chen B, Liu K, Yang L (2020). Sex- and age-specific mild cognitive impairment is associated with low hand grip strength in an older Chinese cohort. J Int Med Res.

[32] Luo H, Li J, Zhang Q, Cao P, Ren X, Fang A, Liao H, Liu L (2018). Obesity and the onset of depressive symptoms among middle-aged and older adults in China: evidence from the CHARLS. BMC Public Health.

[33] Kroenke K, Spitzer RL, Williams JB, Monahan PO, Löwe B (2007). Anxiety disorders in primary care: prevalence, impairment, comorbidity, and detection. Ann Intern Med.

[34] Kroenke K, Spitzer RL, Williams JB (2001). The PHQ-9: validity of a brief depression severity measure. J Gen Intern Med.

[35] Xu WQ, Lin LH, Ding KR, Ke YF, Huang JH, Hou CL, Jia FJ, Wang SB (2021). The role of depression and anxiety in the relationship between poor sleep quality and subjective cognitive decline in Chinese elderly: exploring parallel, serial, and moderated mediation. J Affect Disord.

[36] Wood SN. Generalized additive models: an introduction with R. New York: CRC press; 2006.

[37] Sha T, Cheng W, Yan Y (2019). Prospective association between sleep-related factors and the trajectories of cognitive performance in the elderly Chinese population across a 5-year period cohort study. PLoS One.

[38] Minett TS, Dean JL, Firbank M, English P, O'Brien JT (2005). Subjective memory complaints, white-matter lesions, depressive symptoms, and cognition in elderly patients. Am J Geriatr Psychiatry.

[39] Scheef L, Grothe MJ, Koppara A, Daamen M, Boecker H, Biersack H, Schild HH, Wagner M, Teipel S, Jessen F (2019). Subregional volume reduction of the cholinergic forebrain in subjective cognitive decline (SCD). Neuroimage Clin.

[40] Liew TM (2020). Subjective cognitive decline, anxiety symptoms, and the risk of mild cognitive impairment and dementia. Alzheimers Res Ther.

[41] Jike M, Itani O, Watanabe N, Buysse DJ, Kaneita Y (2018). Long sleep duration and health outcomes: a systematic review, meta-analysis and meta-regression. Sleep Med Rev.

[42] Crowley K (2011). Sleep and sleep disorders in older adults. Neuropsychol Rev.

[43] Pallesen S, Sivertsen B, Nordhus IH, Bjorvatn B (2014). A 10-year trend of insomnia prevalence in the adult Norwegian population. Sleep Med.

[44] Krause AJ, Simon EB, Mander BA, Greer SM, Saletin JM, Goldstein-Piekarski AN, Walker MP (2017). The sleep-deprived human brain. Nat Rev Neurosci.

[45] Klinzing JG, Niethard N, Born J (2019). Mechanisms of systems memory consolidation during sleep. Nat Neurosci.

[46] Spira AP, Gamaldo AA, An Y, Wu MN, Simonsick EM, Bilgel M, Zhou Y, Wong DF, Ferrucci L, Resnick SM (2013). Self-reported sleep and β-amyloid deposition in community-dwelling older adults. JAMA Neurol.

[47] Patel SR, Zhu X, Storfer-Isser A, Mehra R, Jenny NS, Tracy R, Redline S (2009). Sleep duration and biomarkers of inflammation. Sleep.

[48] Kang I, Kim S, Kim BS, Yoo J, Kim M, Won CW (2019). Sleep latency in men and sleep duration in women Can be frailty markers in community-dwelling older adults: the Korean frailty and aging cohort study (KFACS). J Nutr Health Aging.

[49] Park S, Lee S, Kim Y, Lee Y, Kang MW, Kim K, Kim YC, Han SS, Lee H, Lee JP (2020). Short or long sleep duration and CKD: a Mendelian randomization study. J Am Soc Nephrol.

[50] Grandner MA, Sands-Lincoln MR, Pak VM, Garland SN (2013). Sleep duration, cardiovascular disease, and proinflammatory biomarkers. Nat Sci Sleep.

[51] Miller MA, Kandala NB, Kivimaki M, Kumari M, Brunner EJ, Lowe GD, Marmot MG, Cappuccio FP (2009). Gender differences in the cross-sectional relationships between sleep duration and markers of inflammation: Whitehall II study. Sleep.

[52] Lim AS, Saper CB (2011). Sleep, circadian rhythms, and dementia. Ann Neurol.

[53] Leng Y, Musiek ES, Hu K, Cappuccio FP, Yaffe K (2019). Association between circadian rhythms and neurodegenerative diseases. Lancet Neurol.

[54] Dik MG, Jonker C, Comijs HC, Bouter LM, Twisk JW, van Kamp GJ, Deeg DJ (2001). Memory complaints and APOE-epsilon4 accelerate cognitive decline in cognitively normal elderly. Neurology.

